# Investigation of Auxetic Structural Deformation Behavior of PBAT Polymers Using Process and Finite Element Simulation

**DOI:** 10.3390/polym15143142

**Published:** 2023-07-24

**Authors:** Yanling Schneider, Vinzenz Guski, Ahmet O. Sahin, Siegfried Schmauder, Javad Kadkhodapour, Jonas Hufert, Axel Grebhardt, Christian Bonten

**Affiliations:** 1 Institute for Materials Testing, Materials Science and Strength of Materials (IMWF), University of Stuttgart, Pfaffenwaldring 32, D-70569 Stuttgart, Germany; vinzenz.guski@imwf.uni-stuttgart.de (V.G.); st170527@stud.uni-stuttgart.de (A.O.S.); siegfried.schmauder@imwf.uni-stuttgart.de (S.S.); 2 Department of Mechanical Engineering, Shahid Rajaee Teacher Training University, Tehran P.O. Box 16785-163, Iran; javad.kad@gmail.com; 3 Institut für Kunststofftechnik, Universität Stuttgart, Pfaffenwaldring 32, D-70569 Stuttgart, Germany; jonas.hufert@ikt.uni-stuttgart.de (J.H.); axel.grebhardt@h2fly.de (A.G.); christian.bonten@ikt.uni-stuttgart.de (C.B.)

**Keywords:** auxetic behavior, biodegradable, residual stress distribution, warpage, FE-based process simulation, computed tomography

## Abstract

The current work investigates the auxetic tensile deformation behavior of the inversehoneycomb structure with 5 × 5 cells made of biodegradable poly(butylene adipate-coterephthalate) (PBAT). Fused deposition modeling, an additive manufacturing method, was used to produce such specimens. Residual stress (RS) and warpage, more or less, always exist in such specimens due to their layer-by-layer fabrication, i.e., repeated heating and cooling. The RS influences the auxetic deformation behavior, but its measurement is challenging due to its very fine structure. Instead, the finite-element (FE)-based process simulation realized using an ABAQUS plug-in numerically predicts the RS and warpage. The predicted warpage shows a negligibly slight deviation compared to the design topology. This process simulation also provides the temperature evolution of a small-volume material, revealing the effects of local cyclic heating and cooling. The achieved RS serves as the initial condition for the FE model used to investigate the auxetic tensile behavior. With the outcomes from FE calculation without consideration of the RS, the effect of the RS on the deformation behavior is discussed for the global force–displacement curve, the structural Poisson’s ratio evolution, the deformed structural status, the stress distribution, and the evolution, where the first three and the warpage are also compared with the experimental results. Furthermore, the FE simulation can easily provide the global stress–strain flow curve with the total stress calculated from the elemental stresses.

## 1. Introduction

A promising class of materials for structural components is that of metamaterials with auxetic behavior. Auxetic structures have a negative Poisson’s ratio [1], which means that such structures behave counterintuitively. They are generally cellular and are called auxetic materials. Detailed descriptions of Poisson’s ratios are provided in [1,2,3,4,5,6,7,8], which present both the general definition of the Poisson’s ratio and specific deductions of auxetic cells. Most existing auxetic structures are synthetic [9], so-called mechanical metamaterials; however, a few natural auxetic structures exist, e.g., those reported in [10,11,12]. A review of auxetic structures is provided by Kelkar et al. [13] and Negrea [14]. In producing auxetic structures, additive manufacturing (AM) has provided an efficient production process from design to prototype and to final applicable components. This technique also allows for the quick production of complex geometries: 3D printing, for instance, is an example of AM. Some review articles about AM can be found in [15,16,17]. The layer-by-layer production process causes the reheating of underlying materials near the placement of extrusions; moreover, reheated materials also undergo repeated cooling. These heating–cooling cycles, in addition to the contraction between the printed part and the built part, result in residual stresses (RSs) in the structure. In order to obtain knowledge of the RS in the structure after printing, numerical prediction can be utilized, e.g., finite element (FE) simulation. Concerning FE simulation, using the inherent functions of a particular software is an efficient way to predict the material and structural deformation behavior numerically. Advanced users develop their subroutines for executing the simulation, since the provided inherent functions are not sufficient enough to achieve their goals. Sejnoha et al. [18] applied the modified Mori–Tanaka micromechanical model to describe the nonlinear viscoelastic response of unidirectional fibrous composites (made of basalt fabrics bound to an epoxy resin, L285 Havel). Here, the FE-based multiscale approach is preferred.

Warpage, more or less, always exists in auxetic structures manufactured with fused deposition modeling (FDM). Concerning the investigation of warpage, the existing literature includes studies using tests and simulations. Singh [19] experimentally studied the warping of 3D models made of acrylonitrile butadiene styrene (ABS) and polylactic acid (PLA) and broadly classified the solutions into three categories, treating the bed with chemical solutions, providing an enclosure, and modifying the internal structure of the model. In order to minimize the warpage in ABS prototypes built with a low-cost FDM, Kuo et al. [20] designed a chamber in which to maintain the temperature and increase the modeling space. This work also investigated the optimal process parameters for reducing warpage using the Taguchi method. Subsequently, also using the Taguchi method combined with fuzzy comprehensive evaluation, Zhang and Peng [21] optimized the process parameters of FDM by considering two index parameters: dimensional error and warpage. Alsoufi and Elsayed [22] tackled the influence of the nozzle temperature and printing speeds on FDM 3D components (PLA + filament) in order to study and minimize the warpage. Through their experiments, Yu et al. [23] found that two main factors—auxiliary hearing and the raster angle of the FDM process—significantly influence the tensile properties and the warpage of parts. Using FE simulation, Fitzharris et al. [24] investigated the material parameter influence on the warpage of FDM parts made from semicrystalline polymer polypropylene sulfide (PP). They found that the lower the thermal expansion coefficient, the lower the part warpage, however, the thermal conductivity, the heat capacity, and Young’s modulus showed negligible influence on the warpage. Syrlybayev et al. [25] developed a thermal-mechanical model (ANSYS) in order to optimize the warpage introduced by the rapid heating-cooling process within the FDM process. The investigations in the works mentioned above aimed to minimize the warpage, which means the warpage itself was their study goal. The current work uses the FE-based process simulation (ABAQUS) to predict the warpage and RS. Thus, our FE method, considering process parameters and material properties, can also be used to optimize the warpage. Here, however, the warpage in the FE result indicates the dimensional error between the printed specimen manufactured using FDM and the CAD design. For the case of negligible warpage, the geometry of the CAD design can be further used in the subsequent FE simulation with mechanical loading; otherwise, the real geometry reconstructed from tomographic images should be used. In other words, the warpage is just an intermediate result, which determines the geometry applied in the next step of FE simulation. Since the metamaterials mentioned above possess a negative Poisson’s ratio, which leads to their application in various fields, they exhibit high vibration and damping, as well as good acoustic absorption properties. Such structures are optimal for shock compensation and noise suppression. Most auxetic structures are made from plastic and metallic materials. However, more published data exist for the former than for the latter; thus, our investigation aims to experimentally and numerically study the auxetic structural behavior of biodegradable polymers under tension. For the first step, the inverse honeycomb structure is produced using the FDM technique, for which 100% biodegradable polylactide acid (PLA) and poly(butylene adipate-co-terephthalate) (PBAT) are preferred, as their blends achieve the best combination for high strength and good ductility. In order to analyze the tomographic data in detail and automatically, the machine learning method is also applied, the results of which will be reported in our consecutive work. This work is a continuation of Schneider et al. [26], emphasizes the FE-based processing simulation used to predict the RS numerically, and then consecutively applies the RSs in the subsequent mechanical loading. The effect of RS on auxetic behavior can be presented by comparing the results with and without the consideration of the RS.

The current work is presented in a style similar to that of Schneider et al. [26] because these two works belong to the same investigative project. The results are relatively numerous; thus, for the sake of brevity and clarity, they are presented in two separate reports.

In the current work, PBAT, which is commercially available, is preferred as the sample material. The aim is to investigate the tensile deformation characteristics of auxetic structures. Experiments and FE simulations, including the FE-based process simulation, are performed. The inverse honeycomb shape, a type of re-entrant structure, is selected. The RS, more or less, exists within the specimen. In this work, RS is predicted using an FE-based process simulation, which is realized through an ABAQUS plug-in “AM Modeler” [27,28]. This simulation enables the consideration of the RS in the FE simulation for subsequent tensile tests. Refer to Hufert et al. [29] and Schneider et al. [26] for detailed descriptions of the experiments. A comparison is thereafter performed between the experimental and numerical results, both with and without consideration of the RS.

## 2. Materials and Experimentation

Commercial PBAT Ecoflex F Blend C1200, a fully biodegradable plastic, was purchased from BASF, Ludwigshafen, Germany. A schematic illustration of the PBAT’s chemical structure is referenced in [30,31], and a detailed description of the material is presented in the works of Hufert et al. [29] and Schneider et al. [26].

The tensile flow behavior of a standard specimen was used in order to calibrate the FE-simulated stress–strain behavior in searching for a suitable material model (theory) with which to predict PBAT deformation behavior. The tensile specimen was printed according to the DIN EN ISO 527-1 standard. Generally, the same test was repeated no less than five times. The experimental results [26,29], Young’s modulus, yield stress *R_p_*_0.2_, ultimate tensile strength and strain, and stress–strain flow behavior were comparable with those found in the literature [32,33]. The mean value of all measured data was used for comparison with numerical predictions in this work. Furthermore, both measurement [29] and FE simulation [26] found that an auxetic structure with 5 × 5 cells is the optimum structure, among 3 × 3, 5 × 5, and 7 × 7 structures, with which to represent auxetic behavior well. Moreover, this selection of 5 × 5 cells is material independent, meaning that the experimental and numerical studies emphasized structures with 5 × 5 cells. The dimensions of a unit cell and this 5 × 5 structure are presented in [26,29]. The results of auxetic structural deformation behavior, such as the force–displacement curve, deformed status according to loading, calculation methods of Poisson’s ratio, and the evolution of Poisson’s ratio, are presented in [26]. In the case of no other specification, all of the tests were performed at the IKT Institute, University of Stuttgart, Germany.

In-situ micro-computed tomography (*µ*CT) was performed at RIF e. V. Dortmund, Germany. The tests were carried out with the v |tome| × L 240 (GE Sensing & Inspection Technologies GmbH, Wunstorf, Germany). For PBAT, the test parameter was 120 keV and 80 µA with a voxel size of 25 µm. Limited by the vertical space of the testing rig and PBAT’s extra high tensile deformation ability, an auxetic specimen with 3 × 3 cells was used. Four scans were performed at loadings of 0, 5, 10, and 15 mm. Hufert et al. [29] presented more detailed descriptions of the test and measured results.

## 3. Process and FE Simulation

In order to numerically predict auxetic structural deformation behavior, FE calculations covering cases of the (printing) process and mechanical loading simulation are performed. The former delivers the warpage and RSs for the latter. Practically, the RS is mapped to the mechanical loading simulation, and the warpage is omitted, since it is negligibly small. The RS is estimated to be low, around 1 MPa. Here, a numerical calculation of the RS is conducted using the process simulation, which is realized with an ABAQUS plug-in called “AM Modeler” [28]. It employs the toolpath–mesh intersection module and a few subroutines to assist the calculations [27] and provides an additional interface with which to define new parameters to set up the simulation. The user interface is subdivided into three parts: data setup, model setup, and simulation setup. The event series and table collection are defined in the data setup part, as well as list time, location, and power values, representing the printing path. The event series originated from G-code, a programming language widely used for various manufacturing machines [34]. The machine controller reads the G-code in order to regulate the motors for their moving direction and velocity [34]. Section 3.1 describes the process simulation steps in more detail, as well as the model used in this work. The model set-up for the mechanical FE simulation is provided by Schneider et al. [26]. Here, only a brief introduction is provided, in Section 3.2.

### 3.1. Simulation of Additive Manufacturing Using FE Method

#### 3.1.1. Approach of Additive Manufacturing Process Simulation

The advantages of AM simulations include predicting RSs in parts, minimizing differences between the part design and the manufactured part, and numerical evaluation of part performance under real load conditions. ABAQUS offers the toolpath–mesh intersection function for AM simulations. This function enables the detection of geometric intersections between a toolpath and the FE meshing of a part to be manufactured.

ABAQUS has two methods for simulating AM processes: a thermomechanical and an eigenstrain-based simulation. The latter has been successfully used to evaluate RSs from welding processes. The results obtained with this method are usually more accurate compared to those obtained with thermomechanical simulation. The main drawback of the latter is that more effort is required in order to calibrate the eigenstrain values, either through experimentation or by running other simulations.

The thermomechanical simulation requires a separation of the simulation into two parts: a heat transfer analysis is performed using a thermal model first, and then a stress analysis is conducted using a mechanical model to calculate the deformations in the component and the stress distribution. The heat transfer analysis delivers the evolution of the temperature distribution based on a moving heat source and thermal boundary conditions, such as convection, conduction, or radiation. This temperature distribution, presented as the temperature at each node in every time increment, is then transferred to the stress analysis as a predefined field. Finally, the deformations and stresses can be calculated with the mechanical constraints. With this method, it is possible to include machine information and process parameters, such as laser power, layer thickness, toolpath, support structures, and the substrate on which the part and the support are built, in order to evaluate their influences on thermal behavior, part distortions, and residual stresses.

**Sequential thermomechanical simulation of the additive manufacturing process**. The AM simulation must include the typical characteristics of the 3D printing process: a progressive material deposition, a progressive heating of the deposited material, and a progressive cooling of the printed part. The progressive material deposition is modeled using the progressive element activation function provided, using ABAQUS/Standard [27]. Elements with this characteristic can be either partially or completely filled with material or simply remain empty, i.e., inactive. The material deposition is defined through a so-called “event series”, which defines the time and space coordinates. Moreover, event series also defines the objective of controlling the amount of material added to an element within a particular time increment.

**Thermal Analysis**. The conducted three-dimensional heat transfer analysis is governed by the heat transfer energy balance, which is written as follows:

(1) $$\rho C_{p}\frac{\partial T}{\partial t}=-\nabla q(r,t)+Q(r.t)$$

where *ρ*, *C_p_*, *T*, *t*, *Q*, and *r* are the material density, the specific heat capacity, the temperature, the time, the heat source, and the relative reference coordinate, respectively. *q* is the heat flux vector, calculated as follows:

*q* = −*k*∇*T*, (2)

where *k* is the thermal conductivity of the material. The thermal condition on the boundaries is described by the mechanism’s radiation, conduction, or convection. The first mechanism depends on the surface quality and the color, which affect emissivity. Radiation and convection are due to heat loss into the environment via the component’s surface. Conduction is observed if the component is in contact with another component, which could be a substrate or fixation to position the component. By comparison, convection depends on the surrounding medium and the flow behavior of this medium. The heat flux on a surface due to convection is governed by:

*q* = −*h*(*T* − *T*_0_), (3)

where *h* is the coefficient of convection and *T*_0_ is the ambient temperature. Due to missing data, in order to assign a certain amount of heat loss on a surface from radiation, the effect of radiation is taken into account as a part of convection.

In the process simulation, a moving heat source represents the laser or heat source, which assigns a calculated heat flux to a defined volume. Such a heat flux distribution within a defined volume can be calculated using different formulations. In the past, heat source models introduced the heat at the surface of the component, such as the circular disc model proposed by Pavelic et al., which describes a Gaussian surface flux distribution on the surface of a component [35]. Further investigations led to the development of a hemi-spherical power density distribution or ellipsoidal power density distribution with which to introduce the heat within a volume.

In ABAQUS, three different factors can define the shape of the moving heat source. The first factor is that of a moving concentrated heat flow. This is recommended when the sizes of the finite elements are significantly larger than the size of the laser spot. When the laser spot’s size is comparable to the element’s size, it is recommended to specify the moving heat source with a so-called Goldak distribution or with a uniform distribution. The first is a double ellipsoidal power density distribution often used in welding simulations. The second distributes the laser power uniformly over a box-shaped volume. Thereafter, the volumetric heat power *P_vol_* is calculated as follows:

(4) $$P_{vol}=\frac{\eta P_{laser}}{vwh}$$

where *η* is the absorption coefficient, *P_laser_* is the laser power, *v* is the scan speed of the AM process, *w* is the width of the box-shaped volume, and *h* is the height of the box-shaped volume. The heat source is then defined by the basic geometry. The absorption coefficient *η* depends on the optical properties of the material, the laser wavelength, and the surface temperature [36]. For the FDM process, the nozzle represents the laser, which extrudes the molten filament. In this case, the absorption coefficient is assumed to be 1.

**Mechanical analysis**. Stress analysis requires the set-up of a mechanical model first. Thereafter, the stresses and the deformations are determined by performing a three-dimensional quasi-static incremental analysis. Finally, the build plate needs to be constrained to represent the experimental conditions and avoid rigid body movement. The mechanical analysis is performed under the consideration of a quasi-static equilibrium state. The contact between the build plate and the printed part is assumed to be ideal. The mechanical load or thermal stress *σ_th_* results from the applied temperature evolution and the different thermal expansion coefficients of the build plate material and the printed material, which is obtained via:

*σ_th_* = *Eα*(*T* − *T*_0_), (5)

where *E* is the Young’s modulus and *α* is the thermal expansion coefficient. Plastic deformation at elevated temperatures, accompanied by the reduced yield strength compared to room temperature, results in RSs contained in a solid material despite the absence of external forces. The RSs can be either tensile or compressive at a particular point. Generally, a component contains a combination of both stresses because the sum of all RSs over the whole component must be zero.

#### 3.1.2. Current Process Simulation

Components manufactured with the fused filament fabrication (FFF) process contain warpage and RSs due to layer-by-layer building and thermal differences in the volume [37]. Additionally, materials with a high coefficient of thermal expansion are prone to experience more deformation during the AM process [38]. Hence, the material model in process simulation needs various properties to be defined. The properties defined for this study are density, elasticity, plasticity, specific heat, thermal expansion, and thermal conductivity. Refer to [27,28,39] for a more detailed description of the process simulation.

**Figure 1:**
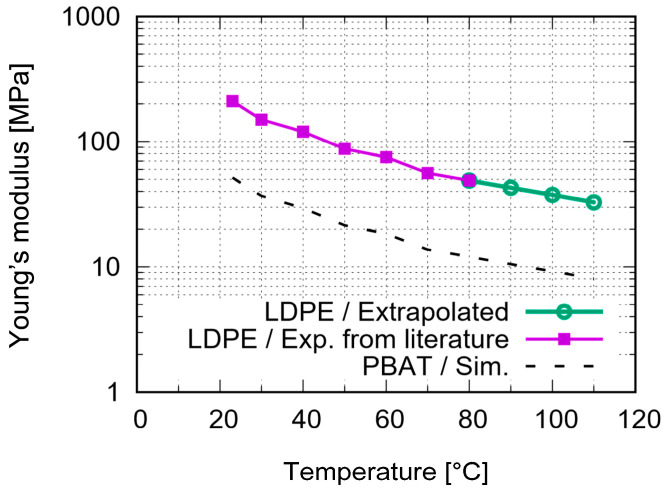
Temperature dependent LDPE Young’s modulus values for elasticity model [43] and the estimated values for PBAT [43].

**Figure 2:**
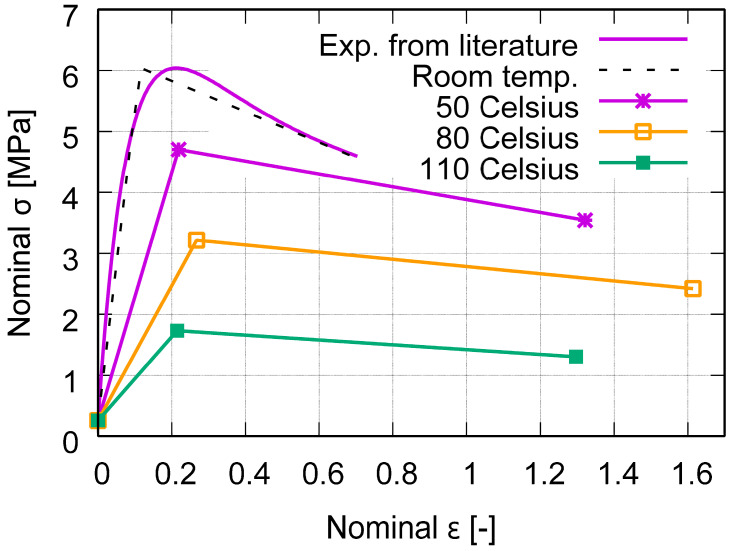
Estimated temperature-dependent nominal stress–strain behavior for PBAT [43], where the linear behavior is the request of the ABAQUS plug-in [27].

**Figure 3:**
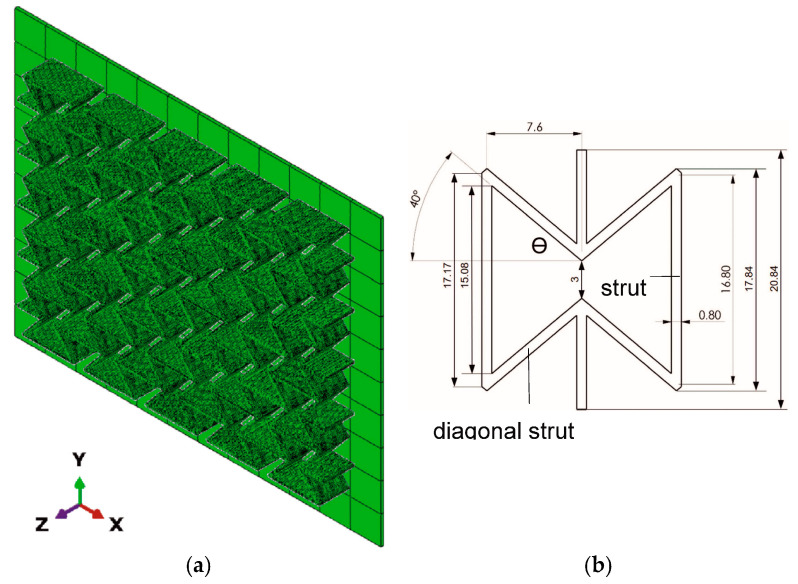
(**a**) Meshing used in the process simulation and the plate at 60 °C, where the meshing of the auxetic structure is identical in the FE simulation and in the process simulation; and (**b**) the dimensions of the unit cell (in mm) [26].

The melting point of PBAT is 110–120 °C [33,40]. Due to the available data for PBAT being limited from the current investigation and the literature, it is challenging to find its data at elevated temperatures. Thus, some of the required input data were interpolated with the help of low-density polyethylene (LDPE) data for higher temperature regimes since PBAT and LDPE behave similarly under loading. LDPE is also a semi-crystalline polymer with a melting point of approximately 110 °C [41,42] and a glass transition temperature of −32.2 °C [41], which are comparable to the PBAT data. The Poisson’s ratio is assumed to be 0.41 for the entire temperature range. Figure 1 presents the temperature dependent Young’s modulus for LDPE [43] and the estimated values for PBAT. These data are used in the current work. Figure 2 denotes the estimated nominal stress–strain behavior at different temperatures, where the linear behavior fulfills the requirements of the ABAQUS plug-in mentioned above. The endpoints of the increasing part of each curve in Figure 2 correspond to the yield stresses at the particular temperature. Concerning the FE-based process simulation, Figure 3 illustrates (a) the meshed plate and the auxetic structure, and (b) the dimensions of the unit cell [26]. The plate maintains a constant temperature of 60 °C during printing. The meshing of the auxetic structure is identical to that used in the mechanical loading.

### 3.2. FE Simulation with Mechanical Loading

**Figure 4:**
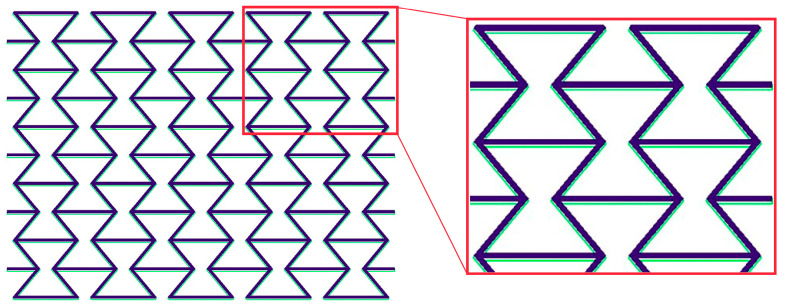
Overlay view for the initial and post-cooling state, with warpage of the PBAT auxetic structure for 5 × 5 cells: green contour represents the initial state and dark purple area represents the state after cooling to room temperature of 23 °C.

Using ABAQUS [27,44] inherent functions (theory/material models) to predict polymer deformation behaviors is preferred. Schneider et al. [26] found that the “Ogden” model, with *n* = 4, is suitable for predicting the PBAT deformation behavior. There are a total of 738,760 octagonal and hexagonal elements of types C3D8H and C3D6H. Detailed descriptions of the selection and a brief review of the FE simulation as applied for polymers are presented by Schneider et al. [26]. The current work follows Schneider et al. [26,45], in that the “Ogden *n* = 4” model was used. As mentioned, the predicted warpage caused by the printing process is negligibly small and, for simplicity, the current work used the initial CAD-design geometry, which is identical to the meshed structure applied for process simulation (Figure 3). Figure 4 illustrates the overlay view of the initial CAD design geometry and the predicted deformed state after cooling, where the green contour represents the initial state and the dark purple area shows the state at room temperature 23 °C, after cooling. Homogenous boundary conditions (BCs) are used. The initial state considers the RS distribution calculated with the process simulation.

## 4. Comparison of Simulated Results with Test Data

### 4.1. Numerical Results Predicted with Process Simulation

**Figure 5:**
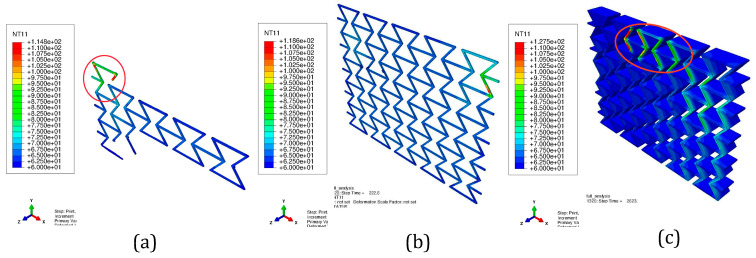
Temperature distribution during the process simulation: (**a**) at the beginning of printing; (**b**) when the first layer is completely printed; and (**c**) when the whole structure is printed.

The printed materials experience reheating-and-cooling processes during printing, especially when additional material is deposited. Figure 5 illustrates the temperature distribution while the printing process. Figure 5a presents the status during printing the first layer.

**Figure 6:**
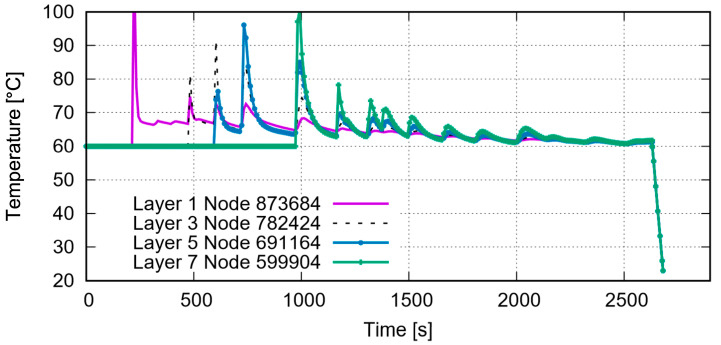
Nodal temperature evolution over time for the nodes with same (X, Y) positions in different layers.

**Figure 7:**
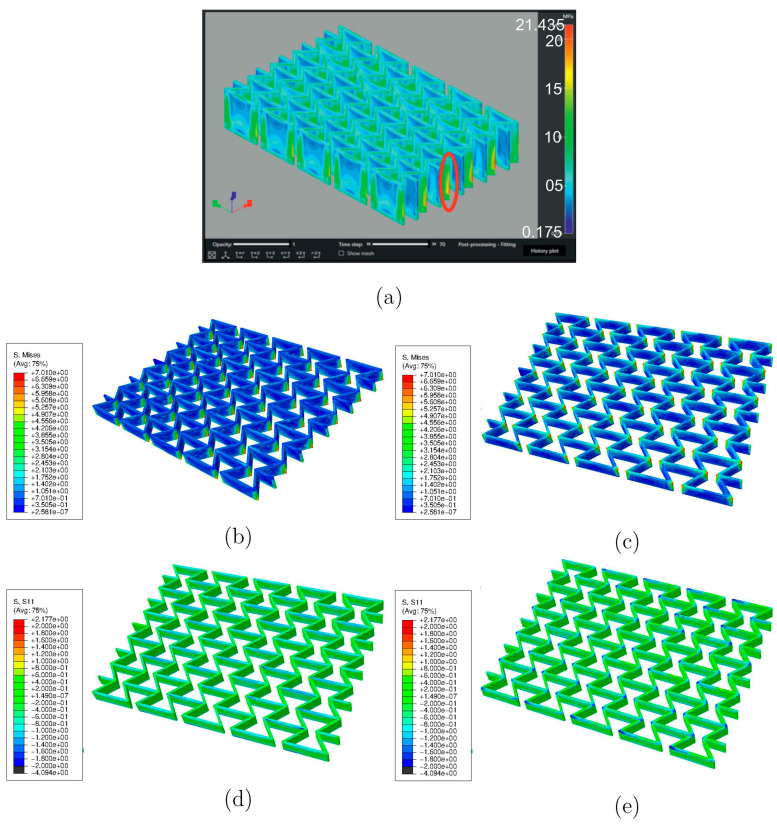
The process-simulation-predicted RS distribution after printing and cooling down to room temperature: (**a**) using Digimat-AM [46] with ABS as the sample material and (**b**–**e**) using the ABAQUS plug-in [27,28] with PBAT, where (**b**,**c**) and (**d**,**e**) are presented as von Mises and X-direction (loading direction for further tension) stresses.

Not a single layer has been completely printed yet. The solid circle marks the place where the just-printed material still shows a high temperature. The material marked with the dashed circle has already cooled down to or very near to the plate temperature of 60 °C. Figure 5b,c demonstrates the printed status at which the first layer or the whole structure is completely printed, respectively. As mentioned in Section 3.1, the temperature is evaluated at each node in the process simulation. In order to show the temperature evolution in detail during the printing process, Figure 6 presents the nodal temperature time history for four nodes, on top of layers 1, 3, 5, and 7. The maximum temperature during printing is around 100 °C. One essential factor influencing the further deformation behavior of the specimen is the RS. Its distribution is provided in Figure 7 after-cooling to room temperature.

**Figure 8:**
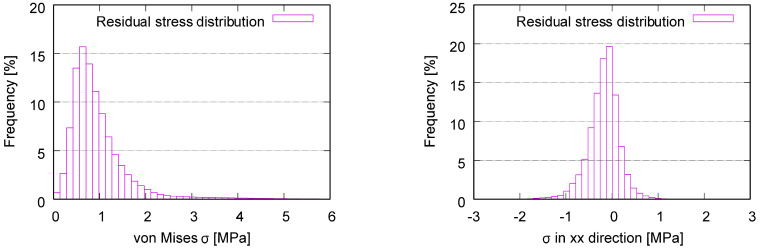
Histogram of RSs after cooling, predicted with process simulation: (**a**) von Mises stress with a mean value of 0.984 MPa and (**b**) stress in the X-direction.

**Figure 9:**
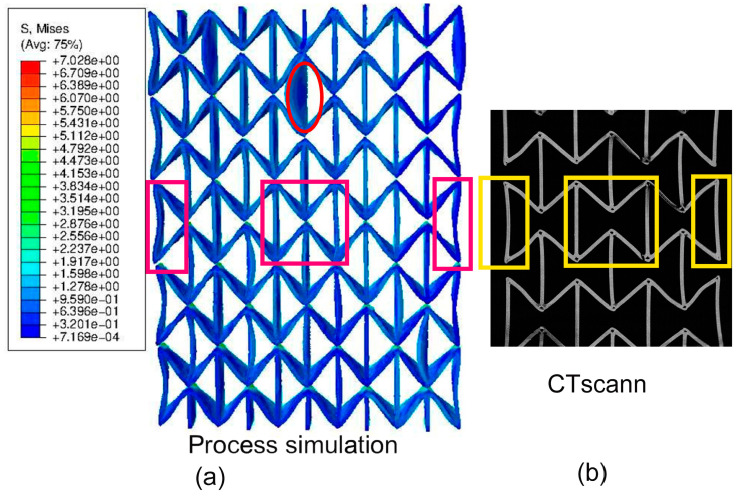
Warpage comparison between experiment and simulation as initial status for tensile loading: (**a**) from the process simulation shown as the first printed layer on the outer side with a zoom factor of 5 for the warpage and (**b**) a selected CT scan image nearest to the surface from a sample with 3 × 3 cells.

Figure 7a results from another process simulation using software Digimat-AM (version 2019.0) [46] for a sample material of acrylonitrile butadiene styrene (ABS) [47]. The oval marks the region with relatively high RSs. This subfigure is used to prove that the RS distribution predicted using the ABAQUS AM-Modeler is accurate since both process simulations show similar distribution patterns. Figure 7b–e shows results predicted using the ABAQUS plug-in [27,28] for specimens made of PBAT, where Figure b,c and Figure d,e are presented as von Mises and X-direction (loading direction in further tensile loading) stresses. Figure 8 illustrates the histogram of RSs after cooling, as predicted with the process simulation. Figure 8a shows von Mises stress with a mean value of 0.984 MPa and Figure 8b shows stress in the X-direction with a mean value of −0.167 MPa. The repeated heating–cooling cycles in the printing process would also introduce warpages aside from the RSs. Figure 9 compares the warpage between the predicted and measured results, where the warpage in Figure 9a is shown in an enlarged view with a deformation scale factor of five. As mentioned in Section 2, the *µ*CT test was performed on a PBAT specimen with 3 × 3 cells due to limited space in the facility. However, it is taken that the numerically predicted warpage, marked in magenta rectangles, and the square in Figure 9a, are still comparable to those marked in yellow in Figure 9b. The warpage distribution is non-homogeneous, as demonstrated within the red oval in Figure 9a, where a strut with relatively high warpage is present.

### 4.2. Numerical Results Predicted with Mechanical Simulation

The auxetic deformation behavior is studied under tension using a structure with 5 × 5 cells made of PBAT. The result comparison between the FE simulation (with and without consideration of the RS) and the experiment can deliver more detailed information about the structural deformation behavior. Furthermore, the influence of RS on material behavior can be evaluated. The FE prediction, without consideration of the RS, is analyzed in Schneider et al. [26]. The current work presents the FE results, as simulated with consideration of the RS and the comparison to those without consideration of the RS.

**Figure 10:**
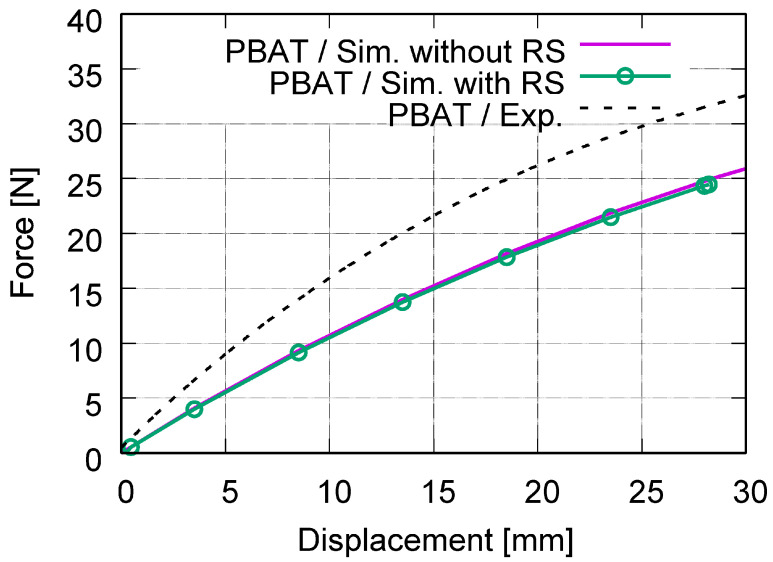
Comparison of the global force–displacement curves achieved from the experiment and FE simulation, with and without [26] considering the RS for the auxetic structure with 5 × 5 cells made of PBAT.

**Figure 11:**
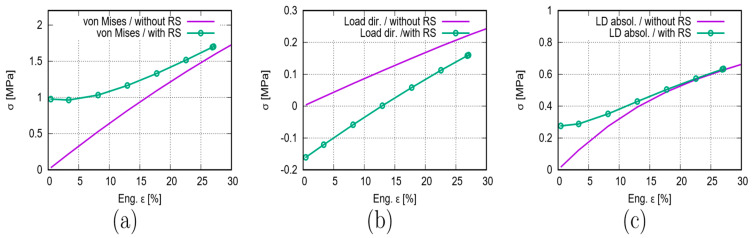
FE-predicted global stress evolution according to loading for a PBAT auxetic structure with 5 × 5 cells: (**a**–c) von Mises, loading direction, and the absolute value of loading direction stress, respectively.

**Figure 12:**
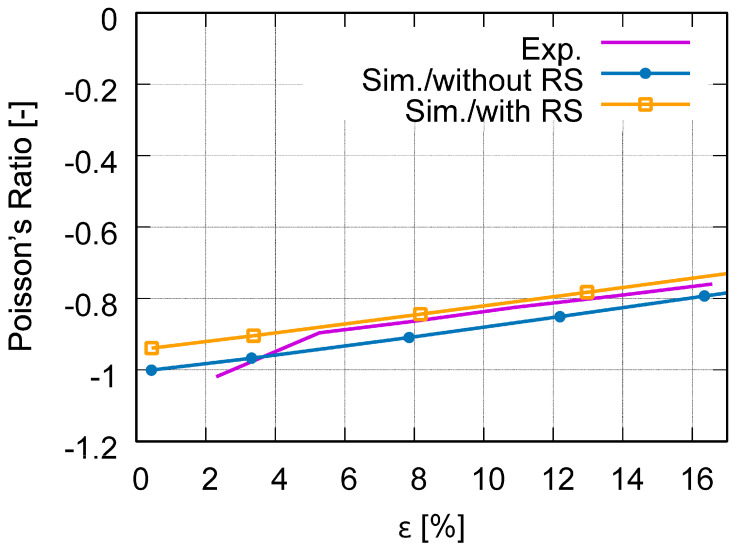
Comparison of the structural Poisson’s ratio evolution according to engineering strain achieved from the experiment and FE simulation, with and without [26] considering the RS for the auxetic structure with 5 × 5 cells made of PBAT.

**Figure 13:**
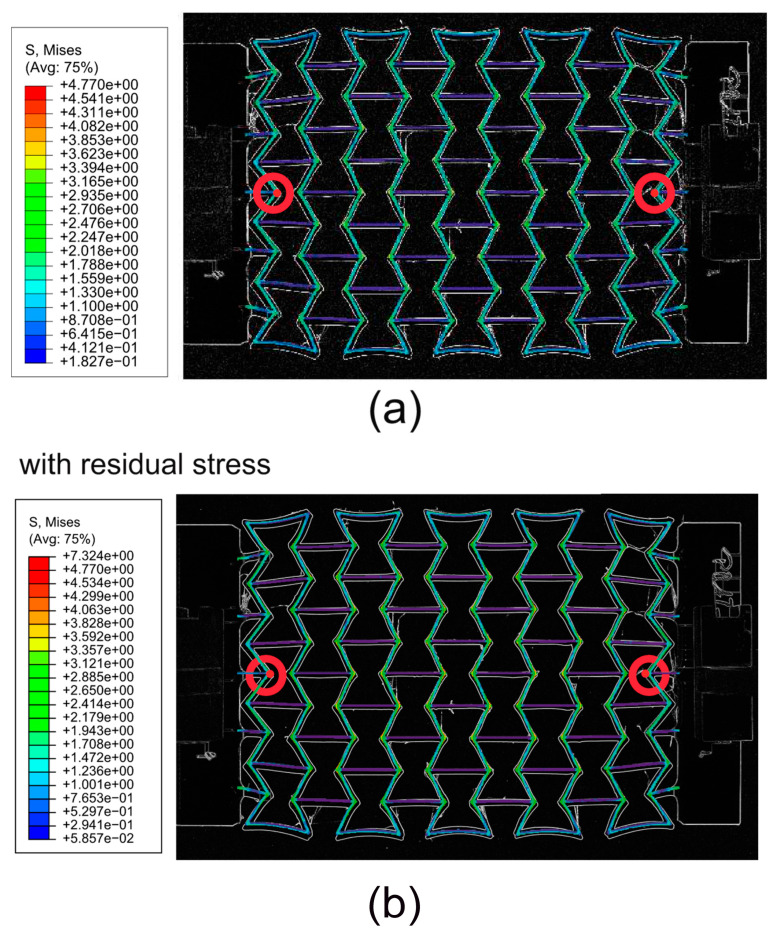
Comparison of PBAT deformed auxetic structures with 5 × 5 cells between experiment (gray contour) and FE simulation (colored area), where the legend only presents the FE result for the von Mises stress distribution: (**a**) FE simulation without RS and (**b**) FE simulation with RS.

**Figure 14:**
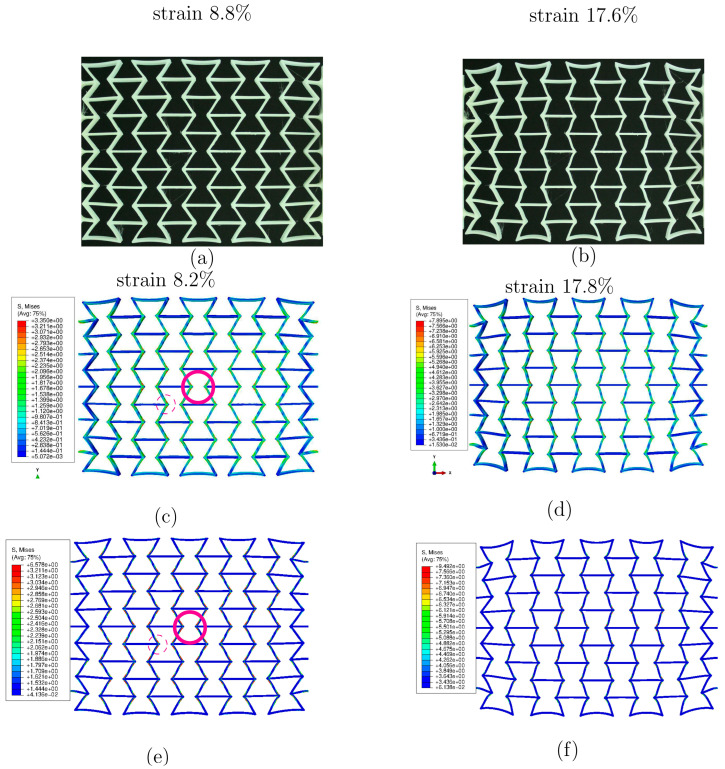
Experimentally measured and numerically predicted deformed status according to loaded engineering strain for a PBAT auxetic structure with 5 × 5 cells: (**a**,**b**) measured at 8.8% and 17.6% strain, respectively; (**c**,**d**) FE-predicted von Mises stress, at 8.2% and 17.8% strain, respectively, without consideration of the RS; (**e**,**f**) FE-predicted at 8.2% and 17.8% strain, respectively, considering RS.

Figure 10 plots the force–displacement curves from the test and from the FE prediction with and without [26] considering the RS. Figure 11 illustrates the global stress evolutionary behavior according to the loading (engineering) strain. The global stress is calculated based on the elemental stresses, considering the weighting factor of the element volume fraction. Figure 11a–c present the evolution of the von Mises, the loading direction, and the absolute value of loading direction stress. According to loading (engineering strain), Figure 12 illustrates the structural Poisson’s ratio evolution obtained from the measurement and simulation, both with and without consideration of the RS for the auxetic structure with 5 × 5 cells. This calculation considers only the middle three rows for the experimental and numerical results since the auxetic deformation of the neighboring rows of clamping jaws is affected by the BCs too much to show accurate auxetic behavior [26]. Figure 13 shows the overlay view of the deformed auxetic structures from the experiment and the FE simulation, where the gray contour presents measured data and the colored area the simulated ones. The legends in Figure 13 are only valid for the FE results. Figure 13a compares the measured and FE predicted deformed status without consideration of the RS, where the distance between the two nodes marked in red is 99.8 mm in the simulation and 100.8 mm in the experiment. For the case of FE simulation with consideration of the RS, the results are shown in Figure 13b analogously. The experimentally measured and numerically predicted deformed statuses are provided in Figure 14 for a PBAT auxetic structure with 5 × 5 cells. Figure 14a,b are measured statuses at 8.8% and 17.6% loading strain, respectively. Figure 14c,d show the FE-predicted von Mises stress at 8.2% and 17.8% strain, respectively, without consideration of the RS, while Figure e,f present FE-predicted von Mises stress at 8.2% and 17.8% strain, respectively, considering RS.

**Figure 15:**
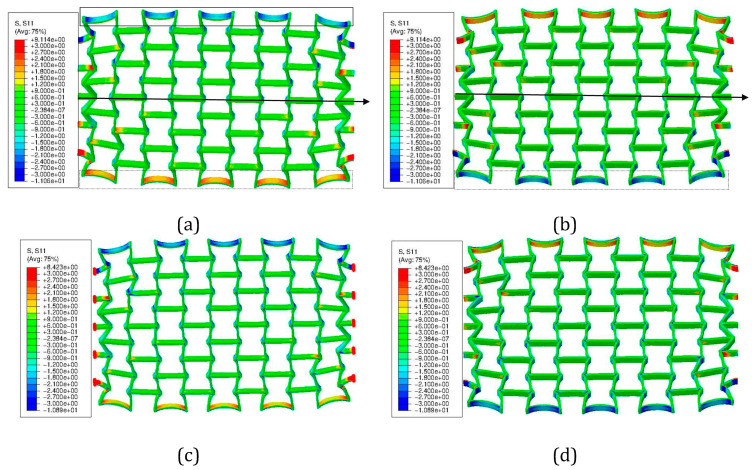
FE-predicted stress distribution in the loading direction for a PBAT auxetic structure with 5 × 5 cells: (**a**,**b**) without consideration of the RS at 27.35% engineering loading strain at two different view aspects; and (**c**,**d**), same as (**a**,**b**), but considering RS at 27.07% strain.

**Figure 16:**
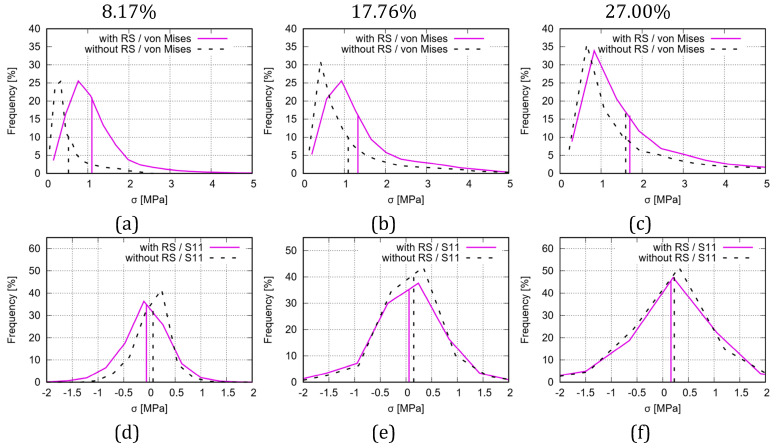
Histogram of FE-predicted stress from simulations with and without consideration of RSs in a deformed auxetic structure with 5 × 5 cells at loading (engineering) strains: (**a**–**c**) von Mises stress at 8.17%, 17.76%, and 27% (without RS 27.35% and with RS 27.07%), respectively; (**d**–**f**) same as (**a**–**c**), but for loading direction stress.

Similar to von Mises stress (Figure 14c,e), the loading direction stress distribution is plotted in Figure 15 at different (engineering) loading status. Figure 15a,b present the deformed status at 27.35% strain with two perspective views, where the RS is not considered. Figure 15c,d are similar to Figure 15a,b, but considering the RS. Figure 16 illustrates the histogram of FE-predicted stress from simulations with and without consideration of RS in the deformed auxetic structure with 5 × 5 cells at various loading (engineering) strains. Figure 16a,c show the von Mises stress at 8.17%, 17.76%, and 27% (without RS 27.35% and with RS 27.07%), respectively. Figure 16d–f are the same as Figure 16a–c, but for the loading direction stress.

## 5. Discussion

### 5.1. Residual Stress and Warpage

In the current work, the significant results from the process simulation include the evolution and distribution of the nodal temperature and the RS. Here, the von Mises and the X-direction stress are presented. The X-direction corresponds to the loading direction when studying the auxetic structural deformation behavior.

After printing, a material with a small volume, presented as an element in the FE simulation, cools down. This small volume of material will heat its neighbors at the beginning of this cooling process. Furthermore, its neighbors will heat their neighbors. This leads to some regions near the small volume of the just-printed material showing higher temperatures than other regions farther away. This reheating process will be repeated due to layer-by-layer printing. From Figure 5a–c, it is evident that the places (region A) immediately following the printing, e.g., the region marked with a circle with a solid line in Figure 5a, possess higher temperatures around the melting temperature than do other regions farther away from region A. Figure 5b presents that the printing is simulated layer by layer, i.e., no elements in the next layer are activated before the previous layer is entirely printed. The already-printed neighbors (neighboring materials) both in the same layer and in underlaid layers are heated again by the just printed materials, as shown in the region marked with an oval in Figure 5c. Due to this repeated heating, relatively high temperatures are shown within a larger region than the region covered by the just-printed material. Aside from the temperature distribution, it is possible to track the node temperature evolution during the whole (pseudo) printing time (Figure 6). Here, it is assumed that all of the nodes have a temperature no less than 60 °C since the plate’s temperature is set to 60 °C. The four selected nodes are on top of each other (similar Z coordinates according to Figure 5).

Among these four selected nodes, the nodes with smaller layer numbers are printed before those with higher layer numbers. Taking the node in the first layer as an example, its temperature evolution is shown in the purple curve in Figure 6; thus, the corresponding element was printed at about 240 s and had the highest temperature at this printing time point. Thereafter, it quickly cooled down to about 68 °C. Before it could further cool down, its neighboring material in the third layer was printed at the time point of about 500 s, leading to a reheating of the node in the first layer. The purple curve illustrates that a second peak, which is lower than the peak at the just printed status, appears (at about 240 s). Generally, the values of the following temperature peaks for a just-printed node (element). If the current printing position is already far away from the considered node (element in the first layer), then the temperature of the node in the first layer would further decrease, i.e., to lower than 68 °C and converging to the plate temperature of 60 °C. The whole printing process took 2631 s (pseudo time). After this time, the entire printed auxetic structure cooled down to room temperature, 23 °C.

After cooling down to room temperature, no testing data are available for the residual stress, about which the simulation can provide some basic knowledge. The FE-predicted residual stress is presented in Figure 7 and Figure 8, and the warpage in Figure 9.

Figure 7a shows the predicted RS distribution (von Mises) for ABS after printing and cooling down, where the software Digimat-AM (version 2019.0) is used. One characteristic of this distribution that it is non-homogeneous and the other is that the region marked with an oval in Figure 7a, at the junction of two inclined struts, possesses a relatively high temperature. Still, the regions (with relatively low Z coordinates) near the plate possess slightly higher RSs than those far away from the plate. One reason could be that the plate hinders the heat transfer from the printed structure to the environment. The RS distribution (von Mises) for the PBAT specimen in Figure 7b is from the prediction obtained by applying the ABAQUS plug-in AM-Modeler. Figure 7b catches all of the above-mentioned three characteristics of the RS distribution in Figure 7a; therefore, the Digimat-AM and AM-Modeler deliver similar results. Figure 7c is identical to Figure 7b, albeit presented in another perspective view. By comparing Figure 7b,c, it is obvious that the region near the plate demonstrates higher RS. Figure 7d,e, the results predicted with the AM-Modeler, present the X-direction RS distribution at two different perspectives. Generally, both positive and negative residual stress exist in the structure for any particular component in the stress tensor, as presented in Figure 7d,e. Figure 8a,b illustrates the histograms of the RSs for the von Mises and for the X-direction stress, respectively. The mean value of the von Mises stress (Figure 8a) is about 0.984 MPa, which is very close to the expected value of around 1 MPa. Theoretically, the mean value for the X-direction RS (one component of the stress tensor) should be zero; however, practically, there are always some small numerical errors in FE simulations, since only numerical solutions are presented (no theoretical solutions are available). This is the reason that the mean value in Figure 8b is about −0.167 MPa, rather than 0 MPa.

Figure 9 compares the predicted and measured warpage. Figure 9a presents the same material status as Figure 7b,c, but with a scaling factor of five for the warpage. The specimen with 3 × 3 cells, a cutaway view of which is shown in Figure 9b, possesses larger warpage than others. Normally, the warpage is much smaller than that shown in Figure 9b.

The exact reason for the large warpage (Figure 9b) is unclear. Possibly, this specimen (Figure 9b) is a trial product while testing the best suitable environment for printing and cooling. However, this specimen’s in-situ *µ*CT data enabled a good comparison for the numerical prediction. Another possibility is that the printing process is unstable, which causes abnormal residual stresses and warpages. Here, both the scaled warpage from the numerical result (Figure 9a) and the selection of a specimen with extra-large warpage (Figure 9b) from the experiment aim to obtain a large enough deflection for identification with the naked eye. From Figure 9, the process simulation delivers a predicted warpage well matchable to the measured specimens. Such a good match is shown for the struts of cells located in the middle and on the left and right sides of the whole specimen, which are marked with rectangles in Figure 9a,b. The initially straight bars marked within rectangles (Figure 9b) deformed and turned out to be arc-shaped after cooling. These phenomena are also well predicted with the FE-based process simulation (Figure 9a), where rectangles also mark the simulated result. Moreover, it is also possible that some local areas possess higher warpage than others, and this phenomenon should depend on the element-activation path during the process simulation, so that the order of the activated element positions varies from layer to layer. Different activation sequences locally cause different heating-cooling cycles, leading to non-homogeneous local warpage.

### 5.2. Auxetic Structural Deformation Behavior

With the numerical predicted residual stress at hand, the initial status within the FE model for then simulation of auxetic deformation behavior under tension can be set more realistically. The discussion of the tensile deformation behavior covers the global force–displacement curves, the global stress–strain flow behavior, the structural Poisson’s ratio evolution, the deformed status, and the stress distribution characteristics. Whenever available, the comparison is performed between experimental and numerical results. The latter includes cases both with and without consideration of the RS. In the following, the FE model without consideration of the RS is called Model-NORS, and the model with consideration is called Model-RS.

As mentioned, the force–displacement curve is the preferred method with which to present the macro tensile deformation behavior of auxetic structures with the possibility of comparing FE and testing results. Figure 10 illustrates the comparison of the force-displacement curves between the test and simulations with and without [26] considering the RS. Moreover, the FE-predicted curve considering the RS behaves minimally softer than the curve without. The two curves from Model-NORS and Model-RS show a negligible slight difference. The FE-predicted force–displacement curves catch the experimental flow behavior well (Figure 10) with regards to the non-linearity and flow behavior, even though the numerical curves behave in a softer fashion than the reality at a particular loading. As mentioned, the testing data for the global stress and strain are not available, since the calculated stress using measured force might be too high due to the small cross-section of the auxetic structure. Otherwise, the experimental result might be misleading in presenting the structural strength. In this case, the FE prediction demonstrates superiority since the global stresses can be calculated based on elemental stresses. Figure 11 illustrates a global stress–strain curve for the PBAT specimen with 5 × 5 cells. In Figure 11a, the global von Mises stress starts at 0 MPa and increases non-linearly according to the global engineering strain in the simulation not considering the RS and the stress value reaches 1.4–1.6 MPa at 22.5% strain. For the simulation considering RS, the initial value equals the mean value from the process simulation (Figure 7a, 0.984 MPa), and then, the curve shows a plateau region until about 8% strain. After that, it increases non-linearly, and the increasing ratio shows a significant discrepancy compared to that from the FE model without consideration of the RS. Generally, the von Mises stress level is higher in the Model-RS than in the Model-NORS. However, the two curves approach each other with increasing loading, which means the RS influences the global von Mises stress at early loading more strongly than later in the process. In reality, the warpage also contains a contraction, i.e., compressive stress exits. In order to show this compression, the global loading direction stress is plotted in Figure 11b. The curve calculated from the Model-NORS starts at zero and increases nearly linearly. The curve calculated from the Model-RS starts at the mean value (−0.167 MPa, Figure 8b) of the residual stress and reaches zero MPa at about 13% strain. Generally, this curve (Model-RS) increases for the whole loading range and locates lower than the curve calculated from Model-NORS. Figure 11c illustrates the absolute value of the loading direction stress flow behavior, meaning that the calculated curves in Figure 11b,c are based on the same original numerical data. The curve predicted in Model-RS starts at the mean value (about 0.276 MPa) of the RS for the whole auxetic structure after cooling down, and it locates above the curve predicted in the Model-NORS until about 15% loading strain. Both curves (Figure 11c) increase non-linearly, especially at the beginning of loading. Thereafter, they approach each other, becoming closer and closer from 10% strain onward, and finally meet each other at about 17% strain, from which point, both curves develop nearly identically.

Figure 12 illustrates that the Poisson’s ratio increases linearly according to loading. The value range of the Poisson’s ratio, presented in the experimental curve covers ≈ −1 to −0.76 in the loading range of 2.3% to 16.6% (engineering strain), meaning that the auxetic deformation ability reduces according to increasing loading strain. The Poisson’s value of −0.76 at about 16.6% loading strain indicates that the structure still possesses excellent auxetic deformation potential since this value is still much lower than zero. The comparison of the measured and predicted Poisson’s ratio evolutions of the auxetic structure (Figure 12) shows that the numerical curves of both FE models (Model-NORS and Model-RS) capture the experimental curve well in both linearity and in value range. The numerical curve predicted in the Model-NORS locates slightly lower than the experimental curve, meaning that the prediction presents better auxetic behavior than the reality, since the values in this FE curve are nearer to the optimum value of −1. Since the FE curve from Model-RS is located above the FE curve from Model-NORS, and is nearly at the same position as the measured curve, the consideration of RS improves the numerical prediction. This outcome also implies that the RS negatively influences auxetic deformation behavior.

A further numerical investigation is necessary in order to map the higher gradient in the experiment within the loading range of about 2.3% to 5%. The speculation is that this higher ratio is dependent upon the geometric parameters of the auxetic structure, and the current FE simulation did not include such parameters in the material model (theory).

The deformed topologies predicted in the Model-NORS and the Model-RS at about 12% loading (engineering) strain are very similar, since both FE results match the measured result very well (Figure 13). However, the stress range from the Model-RS (Figure 13, right) covers a larger distance than that from the Model-NORS (Figure 13, left), meaning that the RS obviously influences the stress magnitude and distribution but not much of the deformed topology. Concerning the auxetic behavior, the results from simulations prove that the two rows as neighbors of clamping jaws should be excluded in calculating the Poisson’s ratio or analyzing the auxetic structural deformation behavior, since the deformation of cells in these two rows is strongly influenced by the BCs. Such cells’ deformation is not the same as those in other rows. For a more detailed discussion about their deformed status, refer to Schneider et al. [26].

Figure 14 further compares the deformed status of the tested (Figure 14a,b) and FE-predicted (Figure 14c–f) auxetic structures. The latter (Figure 14c–f) also illustrates the von Mises stress distribution and its covered value range. The FE prediction of both Model-NORS and Model-RS matches the experimental topological deformation characteristics well. Similar to the results shown in Figure 13, cells in the middle three rows deform less than those at the rows at the end. The struts of each cell on structural free edges (e.g., the left and right rows in Figure 14c) experience higher deformation than the other struts. The major reason for this phenomenon should be the BCs. The first strut type mentioned above is free of constraints from neighboring cells. The cells which were neighbors of the clamping jaw deformed significantly, where the rotation and the distortion are apparent. At about 8% loading strain, the stress range is about (0, 3.35) MPa, as predicted in the Model-NORS (Figure 14c), while it is about (0, 6.58) MPa as predicted in the Model-RS (Figure 14e). At about 17.5% loading strain, the maximum stress values are about 7.89 and 9.49 MPa in the Model-NORS and the Model-RS, respectively. The stress difference between the two FE models decreases with increasing loading, which implies that the residual stress influences the structural stress more obviously in the early loading stage. Moreover, joining positions of horizontal and inclined struts (region-tip), e.g., those denoted in magenta circles (Figure 14c,e), present relatively high stress throughout the whole structure and in both simulations. Here, regions with high stress and those with high strain are coincident. Due to geometrical factors, the strain gradient at such places is high, which results in high strain values. Comparing Figure 14c–e and Figure 14d–f, another characteristic is that the stress distribution in Model-RS (Figure 14e,f) shows higher local concentration than that in Model-NORS (Figure 14c,d). Most regions in the former (Figure 14e,f) are presented in blue (indicating very low stress) and small areas near the region-tip present high-stress values (displayed in red). However, in the latter (Figure 14c,d), there are fewer blue regions (with low-stress values) than there are in the former; additionally, the green-yellow regions (those with intermediate stress values) exist. The large local discrepancy of the stress distribution negatively influences the structural loading burden capacity, which leads to the assumption that the specimen considering RS would break before the specimen without. In reality, it can be taken that the specimen with larger residual stress would be broken earlier than that with less, since every specimen is expected to possess RS. Another characteristic of deformation and stress distribution is that both deformation and stress distribution are approximately symmetrical according to the geometrical middle line of the specimen in the tension direction. This is more apparently shown in Figure 15. The loading direction stress distributions in Figure 15a,b (Model-NORS) are identical, albeit shown in different perspective views. The upper struts, marked in the solid rectangle, and the lower struts, marked in the dashed rectangle, show symmetric deformation according to the marked arrow (Figure 15). The stress distribution on the outer surface of the upper struts (marked in the solid rectangle in Figure 15a) is almost the same as that on the outer surface of the lower struts (marked in the dashed rectangle in Figure 15b). The same deformation and stress distribution characteristic is also presented in the Model-RS (Figure 15b,d). From all four images in Figure 15, a further conclusion that can be drawn is that the struts connecting the clamping jaw, the ends of which are denoted with red points in Figure 15c, are critical regions which possess both large deformation and high stress.

Concerning the conclusion that the RS influence on the stress distribution decreases, this is more evident from the histograms of von Mises and loading direction stresses shown in Figure 16. The discrepancy for both the curve position and the mean value of the von Mises stress between the results predicted in the Model-NORS and the Model-RS (Figure 16a–c) reduces according to loading. The discrepancy caused by RS and its reduction causes a diminishing effect of RS on the stress observed in the structure. The mean values of the von Mises stress for the whole auxetic structure are 0.53, 1.01, and 1.60 MPa at 8.17%, 17.76%, and 27.35% loading (engineering) strain, respectively, from the Model-NORS, while these three values are 1.10, 1.33 and 1.70 MPa (1.70 MPa at 27.07% strain), respectively, from the Model-RS. Recalling the RS stress value of 0.918 MPa (von Mises) at the beginning of the loading, its effect is gradually minimized for the structural stress values, but its influence on the stress distribution pattern remains (comparing Figure 14d,f), particularly in that the effect does not vanish as quickly as does the stress value. Analogously, Figure 16d–f present the results for the loading direction stress and show similar characteristics as in Figure 16a–c, but with a smaller discrepancy for the curve position and the stress value.

## 6. Conclusions

The goal of this study was to find the optimal performance of auxetic structures through experimental and numerical investigations, since auxetic materials possess excellent properties. The environmentally friendly polymers PLA and PBAT were preferred. Numerically, Schneider et al. [26] and the current work presented the study process, which can be generally used for the FE simulations of polymer deformation behaviors. The current work investigates the tensile deformation behavior of auxetic structures made of PBAT polymers and emphasizes the RS’s influence on deformation behavior. The FE-based process simulation predicted both the warpage and the RS. The auxetic structure, as reconstructed from *µ*CT images, was used to calibrate the predicted warpage. Since the predicted warpage was negligibly small, it was not considered in further FE calculation. Testing data are unavailable for the RS and the numerical RS serves as the initial condition for the FE model under tension. The FE results from the models, with and without consideration of the RS, were compared. When testing data were available, comparisons were also made between the FE simulation and the experiment.

From the FE-based process simulation and after cooling down, the junctions of the two inclined struts presented higher residual stress than other places within the cell. The measured non-homogeneous warpage among cells or inside one cell was well predicted. Taking von Mises stress as an indicating factor, the histogram of the FE result showed a mean value of the RS slightly less than 1 MPa, which can serve as a reference value when estimating the real residual stress.

Concerning auxetic structural tensile deformation behavior, the force–displacement curve predicted in the FE model when considering the RS showed negligibly softer behavior compared to the model without. Both FE curves captured the highly non-linear and evolutionary behavior of the experimental curve, where the numerical result behaved softer than reality. At the beginning of loading, the RS illustrated a stronger influence on the global stresses (both the von Mises stress and the stress in the loading direction) in the auxetic structure. Starting at the 15% global (engineering) strain, the global stresses predicted in FE models, with and without consideration of the RS, approached each other. The Poisson’s ratio evolution, predicted in both FE models, matched the experimental result well for the auxetic structure. The FE model with consideration of the RS presented an even better mapping of the experimental result. The RS demonstrated a much smaller effect on topological deformation than the stress value and distribution. Overlapping the deformed status of the auxetic structure from the test and that predicted with the FE calculation showed that both FE models can predict the actual auxetic deformation characteristics in detail. The FE results predicted in both models showed that the deformation and stress distribution presented symmetric behavior, according to the geometric middle line paralleling the loading direction. Generally, RS negatively affects auxetic deformation behavior.

## Data Availability

Data are contained within the manuscript.
